# A Comparison of MCC and CEN Error Measures in Multi-Class Prediction

**DOI:** 10.1371/journal.pone.0041882

**Published:** 2012-08-08

**Authors:** Giuseppe Jurman, Samantha Riccadonna, Cesare Furlanello

**Affiliations:** Fondazione Bruno Kessler, Trento, Italy; Sapienza University of Rome, Italy

## Abstract

We show that the Confusion Entropy, a measure of performance in multiclass problems has a strong (monotone) relation with the multiclass generalization of a classical metric, the Matthews Correlation Coefficient. Analytical results are provided for the limit cases of general no-information (n-face dice rolling) of the binary classification. Computational evidence supports the claim in the general case.

## Introduction

Comparing classifiers' performance is one of the most critical tasks in machine learning. Comparison can be carried out either by means of statistical tests [1], [2] or by adopting a performance measure as an indicator to derive similarities and differences, in particular as a function of the number of classes, class imbalance, and behaviour on randomized labels [3].

The definition of performance measures in the context of multiclass classification is still an open research topic as recently reviewed [4], [5]. One challenging aspect is the extension of such measures from binary to multiclass tasks [6]. Graphical comparison approaches have been introduced [7], but a generic analytic treatment of the problem is still unavailable.

One relevant case study regards the attempt of extending the Area Under the Curve (AUC) measure, which is one of the most widely used measures for binary classifiers but it has no automatic extension to the multiclass case. The AUC is associated to the Receiver Operating Characteristic (ROC) curve [8], [9] and thus proposed formulations were based on a multiclass ROC approximation [10]–[13]. A second class of extensions is defined by the Volume Under the Surface (VUS) approach, which is obtained by considering the generalized ROC as a surface whose volume has to be computed by exact integration or polynomial approximation [14]–[16]. As a baseline, the average of the AUCs on the pairwise binary problems derived from the multi-class problems has also been proposed [17].

Other measures are more naturally extended, such as the accuracy (ACC, *i.e.* the fraction of correctly predicted samples), the Global Performance Index [18], [19], and the Matthews Correlation Coefficient (MCC). We will focus our attention to the last function [20], which in the binary case is also known as the 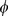-coefficient, *i.e.*, the square root of the average 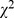 statistic 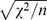 on 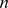 observed samples for the 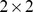 contingency table of the classification problem.

For binary tasks, MCC has attracted the attention of the machine learning community as a method that summarizes into a single value the confusion matrix [21]. Its use as a reference performance measure on unbalanced data sets is now common in other fields such as bioinformatics. Remarkably, MCC was chosen as accuracy index in the US FDA-led initiative MAQC-II for comparing about 13 000 different models, with the aim of reaching a consensus on the best practices for development and validation of predictive models based on microarray gene expression and genotyping data [22]. A generalization of MCC to the multiclass case was defined in [23], also used for comparing network topologies [24], [25].

A second family of measures that have a natural definition for multiclass confusion matrices are the functions derived from the concept of (information) Entropy, first introduced in [26]. In the classification framework, measures in the entropy family range from the simpler confusion matrix entropy [27] to more complex functions as the Transmitter Information [28] and the Relative Classifier Information (RCI) [29]. Wei and colleagues recently introduced a novel multiclass measure under the name of Confusion Entropy (CEN) [30], [31]. They compared CEN to both RCI and accuracy, obtaining better discriminative power and precision in terms of two statistical indicators called degree of consistency and degree of discriminancy [32].

In our study, we investigate the intriguing similarity existing between CEN and MCC. In particular, we experimentally show that the two measures are strongly correlated, and that their relation is globally monotone and locally almost linear. Moreover, we provide a brief outline of the mathematical links between CEN and MCC with detailed examples in limit cases. Discriminancy and consistency ratios are discussed as comparative factors, together with functions of the number of classes, class imbalance, and behaviour on randomized labels.

## Methods

Given a classification problem on 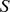 samples 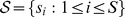 and 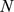 classes 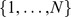, define the two functions 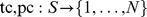 indicating for each sample 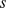 its true class 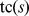 and its predicted class 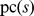, respectively. The corresponding confusion matrix is the square matrix 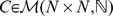 whose 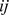-th entry 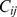 is the number of elements of true class 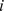 that have been assigned to class 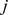 by the classifier:
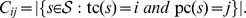
The most natural performance measure is the accuracy, defined as the ratio of the correctly classified samples over all the samples:
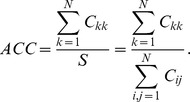



### Confusion Entropy (CEN)

In information theory, the entropy 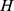 associated to a random variable 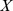 is the expected value of the self-information 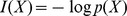:
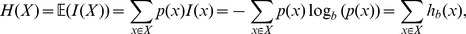
where 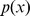 is the probability mass function of 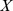, with 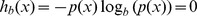 for 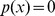, motivated by the limit 
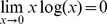
.

The Confusion Entropy measure CEN for a confusion matrix 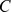 is defined in [30] as:
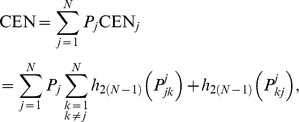
(1)where 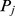, 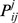, 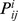 are defined as follows:


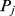
 is the confusion probability of class 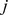: 
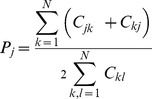



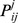
 is the probability of classifying the samples of class 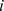 to class 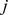 for 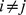 subject to class 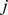:
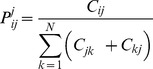


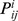
 is the probability of classifying the samples of class 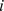 to class 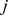 subject to class 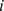:
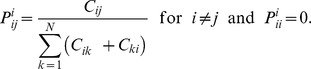



For 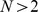, this measure ranges between 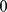 (perfect classification) and 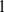 for the complete misclassification case
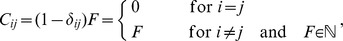
while in the binary case CEN can be greater than 1, as shown below.

### Matthews Correlation Coefficient (MCC)

The definition of the MCC in the multiclass case was originally reported in [23]. We recall here the main concepts. Let 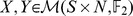 be two matrices where 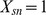 if the sample 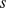 is predicted to be of class 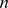 (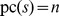) and 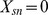 otherwise, and 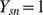 if sample 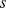 belongs to class 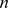 (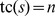) and 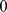 otherwise. Using Kronecker's delta function, the definition becomes:
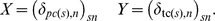
Note that 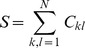, where 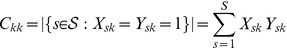, and, for 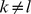, 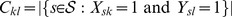.

The covariance function between X and Y can be written as follows:
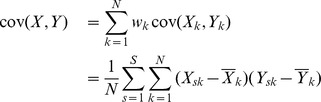
where 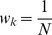 and 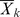 and 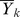 are the means of the 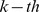 columns defined respectively as 
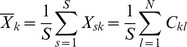
 and 
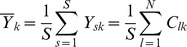
.

Finally the Matthews Correlation Coefficient MCC can be written as:
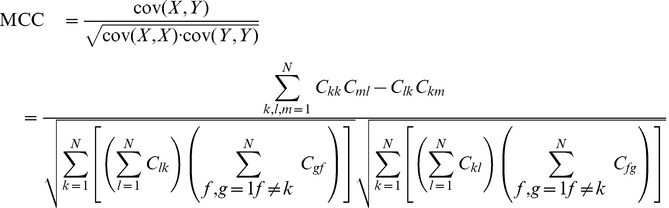
(2)MCC lives in the range 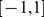, where 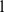 is perfect classification. The value 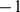 is asymptotically reached in the extreme misclassification case of a confusion matrix 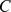 with all zeros but in two symmetric entries 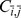, 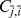. MCC is equal to 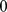 when 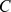 is all zeros but for one column (all samples have been classified to be of a class 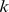), or when all entries are equal 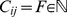.

### Relationships between CEN and MCC

**Figure 1:**
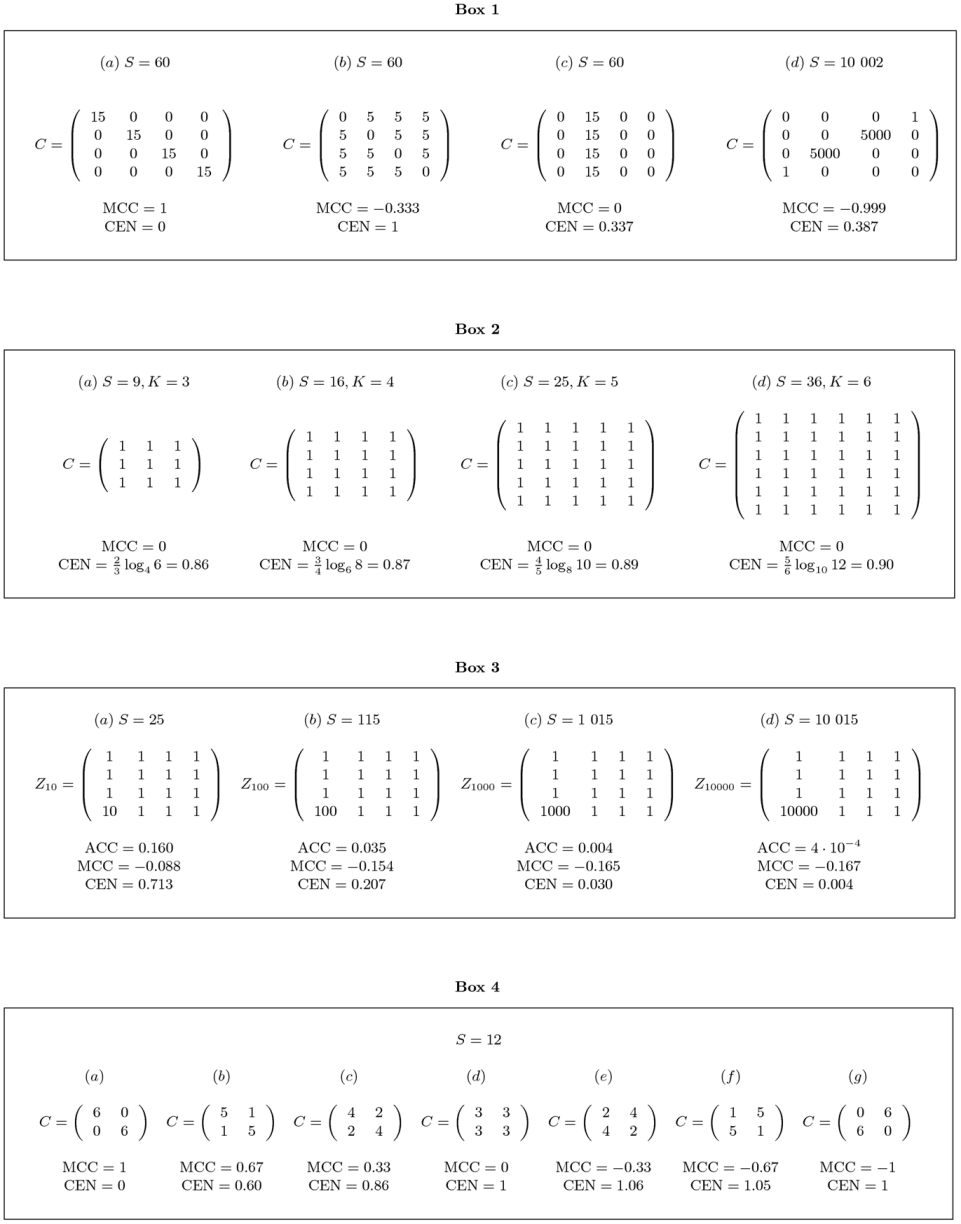
Examples of CEN and MCC for different confusion matrices.

As discussed before, CEN and MCC live in different ranges, whose extreme values are differently reached. In Box 1 of Fig. 1, numerical examples are shown for 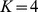 in different situations: (a) complete classification, (b) complete misclassification, (c) all samples classified as belonging to one class, (d) misclassification case in a very unbalanced situation.

It is worth noting that CEN is more discriminant than MCC in specific situations, although the property is not always welcomed. For instance, in Fig. 1, Box 1(c), 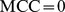 while 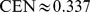. Furthermore, as shown in Box 2, 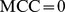 for constant matrix 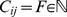 for each 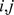, regardless of the number of classes 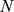, while it is easy to show that 
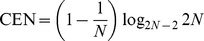
, *i.e.*, CEN is a function of 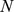. Note that both measures are invariant for scalar multiplication of the whole confusion matrix, so we always set 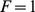 in Box 2.

For small sample sizes, we can show that CEN has higher discriminant power than MCC, *i.e.*, different confusion matrices can have same MCC and different CEN. This can be quantitatively assessed by using the degree of discriminancy criterion [32]: for two measures 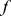 and 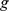 on a domain 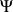, let 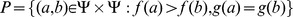 and 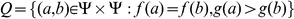; then the degree of discriminancy for 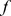 over 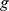 is 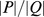. For instance, as in [30], we consider a 3-class case with 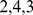 samples respectively: we evaluate all the possible confusion matrices ranging from the perfect classification case
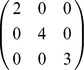
to the complete misclassification case. In this case the degree of discriminancy of CEN over MCC is about 6. Similar results hold for all the 12 small sample size cases on three classes listed in Tab. 6 of [30], ranging from 9 to 19 samples.

We proceed now to show an intriguing relationship between MCC and CEN. First consider the confusion matrix 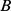 of dimension 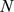 where 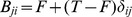, *i.e.*, all entries have value 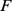 but in the diagonal whose values are all 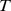, for 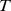, 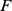 two integers. In this case,
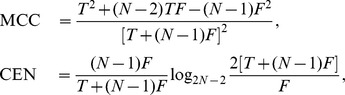
and thus
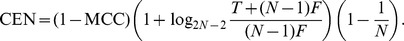
This identity can be relaxed to the following generalization, which slightly underestimates CEN:
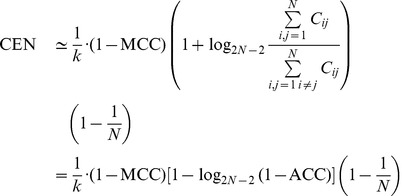
(3)where both sides are zero when 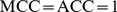, and 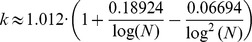. For simplicity's sake, we call “transformed MMC” (tMCC) the right member of Eq. 3.

**Figure 2:**
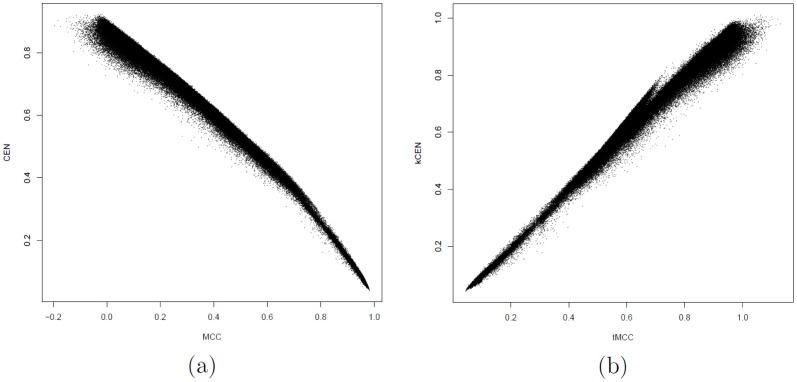
Dotplots of CEN versus MCC. (a) and 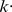
CEN versus tMCC (b) for 200 000 random confusion matrices of different dimensions.

A numerical simulation shows that the tMCC approximation in Eq. 3 holds in a more general and practical setting (Fig. 2). In the simulation, 200 000 confusion matrices 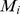 (dimension range: 3 to 30) were generated. For each class 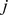, the number of correctly classified elements (*i.e.*, the 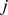-th diagonal element) was uniformly randomly chosen between 1 and 1000. Then the off-diagonal entries were generated as random integers between 1 and 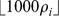, where the parameter 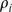 was extracted from the uniform distribution in the range 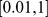, corresponding to small-moderate misclassification. For such data, the Pearson correlation between tMCC and 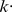
CEN is about 0.994.

In order to compare measures, we consider also the degree of consistency indicator [32]: for two measures 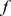 and 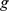 on a domain 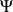, let 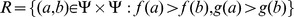 and 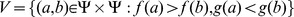; then the degree of consistency 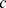 of 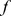 and 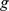 is 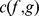 = 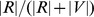. On the given data, 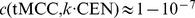, while the degree of discriminancy is undefined since no ties occur. In summary, the relation between tMMC and 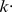
CEN is close to linear on this data, with an average ratio of 1.000508 (CI: 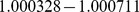, 95% bootstrap Student).

#### Comparison on the 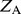 family

The behaviour of the Confusion Entropy is instead rather diverse from MCC and ACC for the family of 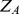 matrices, where all entries are equal but for a non-diagonal one. Because of the multiplicative invariance, all entries can be set to one but for the leftmost lower corner: 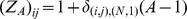 for 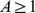 a positive integer. As shown in Fig. 1, Box 3, when 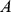 grows bigger, more and more samples are misclassified, *i.e.*, the accuracy 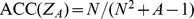 decreases to zero for increasing 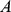.

The MCC measure of this confusion matrix is
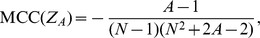
which is a function monotonically decreasing for increasing values of 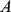, with limit 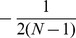 for 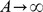.

On the other hand, the Confusion Entropy for the same family of matrices is
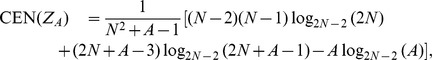
which is still a decreasing function of increasing 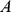, but asymptotically moving towards zero, *i.e.*, to the minimal entropy case. In Box 3 of Fig. 1 we present three numerical examples for 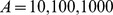.

#### The dice rolling case

Another pathologic case is found in the case of dice rolling classification on unbalanced classes: because of the multiplicative invariance of the measures, we can assume that the confusion matrix for this case has all entries equal to one but for the last row, whose entries are all 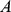, for 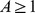. In this case, the Confusion Entropy is
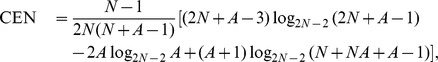
a decreasing function for growing 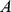 whose limit for 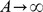 is 
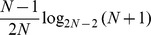
. As a function of 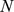, this limit is an increasing function asymptotically growing towards 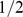. It is easy to see that 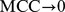 for 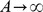 in this case. More in general, while 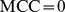 in all those cases where random classification (*i.e.*, no learning) happens, this is lost in the case of CEN, due to its greater discriminant power: there is no unique value associated to the spectrum of random classification problems.

#### The binary case

**Figure 3:**
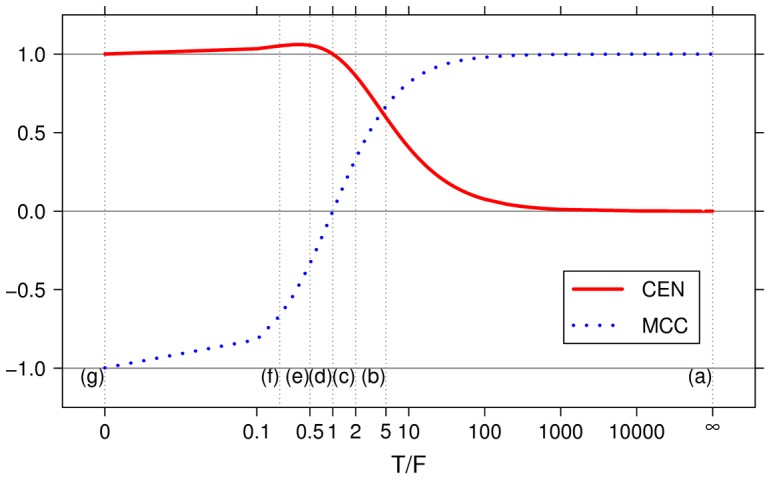
Lines describing CEN and MCC of a confusion matrix 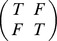 for increasing ratio 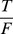. Gray vertical lines correspond to the examples provided in Fig. 1, Box 4.

In the two-class case (P: positives, N: negatives), the confusion matrix is 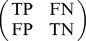, where T and F stand for true and false respectively. The Matthews Correlation Coefficient has the familiar definition [20], [21]:
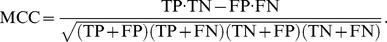



The Confusion Entropy can be written for the binary case as:
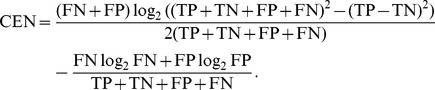
Note that in the case 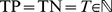 and 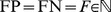, we have
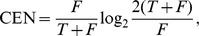
and thus 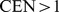 when the ratio 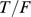 is smaller than 1. In other words, the confusion matrices 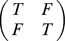 with 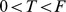 have 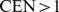; the bound is attained for 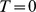, the case of total misclassification. This suggests that CEN should not be used as a classifier performance measure in the binary case. A numerical example is provided in Fig. 1, Box 4, while a plot of CEN and MCC curves for different ratios of 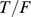 is shown in Fig. 3.

**Figure 4:**
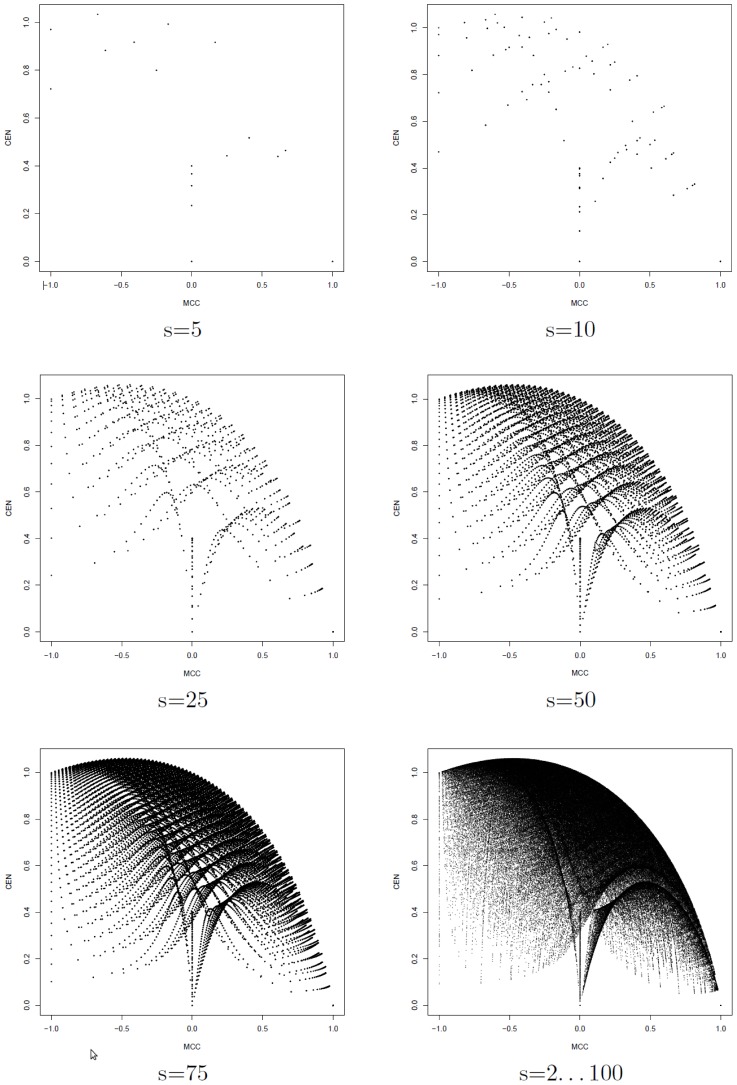
Scatter plots of CEN versus MCC for all the confusion matrices 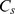 of binary classification tasks with s = 5,10,50,75 samples and for the cumulative set of all 4 598 125 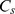 matrices with 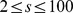.

Indeed, differently from the multi-class case, CEN and MCC are poorly correlated for two classes. We computed MCC and CEN for all the 4 598 125 possible confusion matrices for a binary classification task on 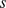 samples (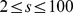). Results are displayed in Fig. 4, for 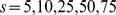 and the cumulative plot with all 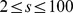. In this last case, the (absolute) Pearson correlation between the two metrics is only 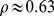.

## Results and Discussion

We compared the Matthews Correlation Coefficient (MCC) and Confusion Entropy (CEN) as performance measures of a classifier in multiclass problems. We have shown, both analytically and empirically, that they have a consistent behaviour in practical cases. However each of them is better tailored to deal with different situations, and some care should be taken in presence of limit cases.

Both MCC and CEN improve over Accuracy (ACC), by far the simplest and widespread measure in the scientific literature. The point with ACC is that it poorly copes with unbalanced classes and it cannot distinguish among different misclassification distributions.

CEN has been recently proposed to provide an high level of discrimination even between very similar confusion matrices. However, we show that this feature is not always welcomed, as in the case of random dice rolling, for which 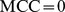, but a range of different values is found for CEN. This case is of practical interest because class labels are often randomized as a sanity check in complex classification studies, *e.g.*, in medical diagnosis tasks such as cancer subtyping [33] or image classification problems (*e.g.*, handwritten ZIP code identification or image scene classification examples) [34].

Our analysis also shows that CEN should not be reliably used in the binary case, as its definition attributes high entropy even in regimes of high accuracy and it even gets values larger than one.

In the most general case, MCC is a good compromise among discriminancy, consistency and coherent behaviors with varying number of classes, unbalanced datasets, and randomization. Given the strong linear relation between CEN and a logarithmic function of MCC, they are exchangeable in a majority of practical cases. Furthermore, the behaviour of MCC remains consistent between binary and multiclass settings.

Our analysis does not regard threshold classifiers; whenever a ROC curve can be drawn, generalized versions of the Area Under the Curve algorithm or other similar measures represent a more immediate choice [35]. This given, for confusion matrix analysis, our results indicate that the MCC remains an optimal off-the-shelf tool in practical tasks, while refined measures such as CEN should be reserved for specific topic where high discrimination is crucial.
